# 602. Usefulness of the DSTM Method in the Era of Drug Resistance

**DOI:** 10.1093/ofid/ofad500.669

**Published:** 2023-11-27

**Authors:** Yuka Yamagishi, Norihisa Nakayama, Akito Doke, Saya Iwame, Yoshie Nishida, Shinji Tokuhiro, Akihito Yokoyama, Hiroshige Mikamo

**Affiliations:** Kochi University, Nankoku, Kochi, Japan; Fukoku CO., LTD, Saitama, Saitama, Japan; Kochi Medical University Hospital, Nankoku, Kochi, Japan; Kochi Medical University Hospital, Nankoku, Kochi, Japan; Kochi Medical University Hospital, Nankoku, Kochi, Japan; Kochi Medical University Hospital, Nankoku, Kochi, Japan; Kochi Medical School, Kochi University, Nankoku, Kochi, Japan; Aichi Medical University, Aichi, Aichi, Japan

## Abstract

**Background:**

Rapid drug susceptibility testing is required as a measure against drug-resistant bacteria. The usefulness of the drug susceptibility testing microfluidic device (DSTM) method, which evaluates drug susceptibility based on morphological changes of bacteria in microfluidic channels, has been reported. We reported that this method can be used as a rapid screening method for ESBL and MBL. In this study, we confirmed the applicability of AmpC to this method. We have also demonstrated the usefulness of this method in directly testing blood cultures with a simple pretreatment. We evaluated blood cultures using this protocol and confirmed its usefulness in a clinical setting.

**Methods:**

We evaluated the ability of DSTM to screen for 84 ESBLs, 24 MBLs, and 43 AmpC strains from clinical isolates with confirmed drug resistance and 156 strains not producing the above enzymes. Furthermore, blood cultures were evaluated using DSTM, and its efficacy was confirmed in clinical practice. A total of 178 specimens that tested positive in the blood culture bottle and were detected as Gram-negative rods by MALDI TOF-MS were evaluated. The specimens were pretreated by two centrifugation procedures to remove blood cell components and evaluated by the DSTM method. For confirmation, resistance genes were evaluated by PCR.

**Figure:**
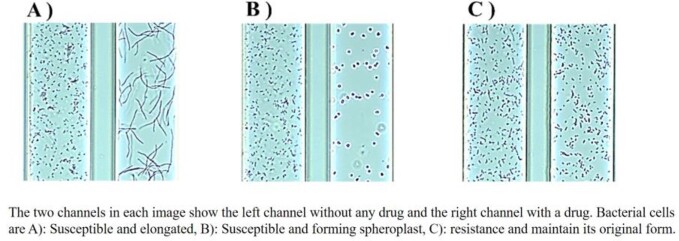

**Results:**

Sensitivity and specificity of ESBL were 95% and 100%, respectively; MBL were 96% and 98%, respectively; AmpC were 58% and 98%, respectively. It was found that the detection methods for chromosomal and plasmid AmpC need to be optimized for each. All specimens evaluated successfully removed blood cell components. Numbers of ESBLs were 21 from *E, coli* and *K. pneumoniae* complex and MBLs were 2 from *M. odoratimimus* using DSTM. 4 specimens containing *Proteus mirabilis* (CTX-M-2) from one patient were false negative for ESBL because those were insensitive to CVA. There were no false positives in the all strains.

**Conclusion:**

As with ESBL and MBL, AmpC could be detected by using morphological changes of the bacteria as an indicator. The pretreatment method for blood culture-positive specimens can confirm the production of β-lactamase within 3.5 hours after a positive blood culture, and is expected to be applied to bacteremia.

**Disclosures:**

**Hiroshige Mikamo, M.D, Ph.D**, Asahi Kasei Pharma Corporation: Grant/Research Support|Merck Sharp & Dohme: Honoraria|Pfizer Inc.: Grant/Research Support|Pfizer R&D Japan: Honoraria|Sumitomo Pharma Co., Ltd.: Grant/Research Support|Sumitomo Pharma Co., Ltd.: Honoraria

